# Supplementary material for: How has COVID-19 changed healthcare professionals’ attitudes to self-care? A mixed methods research study
Source: PLoS One. 2023 Jul 24;18(7):e0289067. doi: 10.1371/journal.pone.0289067 (PMC10365300; doi:10.1371/journal.pone.0289067)
Supplement: S1 Table — (PDF) [file pone.0289067.s001.pdf]

Supplementary Table 1: Resources, competencies, technology and future

RESOURCES AND COMPETENCIES		Doctor	Nurse	Service	Pharmacy	SP/Other	Total	p-value
								0.13
**I have the resources & proficiency to promote self-care in my practice**	Strongly disagree	1 (2.1)	1 (2.3)	2 (4.8)	1 (0.8)	0 (0.0)	5 (1.7)	
	Disagree	6 (12.5)	0 (0.0)	2 (4.8)	15 (12.7)	4 (7.8)	27 (8.9)	
	Neutral	9 (18.8)	7 (15.9)	4 (9.5)	14 (11.9)	8 (15.7)	42 (13.9)	
	Agree	29 (60.4)	27 (61.4)	21 (50.0)	63 (53.4)	25 (49.0)	165 (54.5)	
	Strongly agree	3 (6.3)	9 (20.5)	13 (31.0)	25 (21.2)	14 (27.5)	64 (21.1)	
**My patients are adequately prepared/trained to self-care**	Strongly disagree	4 (8.3)	2 (4.5)	3 (7.1)	6 (5.1)	1 (2.0)	16 (5.3)	
	Disagree	26 (54.2)	9 (20.5)	14 (33.3)	31 (26.5)	16 (32.0)	96 (31.9)	
	Neutral	11 (22.9)	18 (40.9)	15 (35.7)	47 (40.2)	21 (42.0)	112 (37.2)	
	Agree	6 (12.5)	14 (31.8)	8 (19.0)	31 (26.5)	11 (22.0)	70 (23.3)	
	Strongly agree	1 (2.1)	1 (2.3)	2 (4.8)	2 (1.7)	1 (2.0)	7 (2.3)	
**My /patients have the competency & resources to self-care**	Strongly disagree	5 (10.4)	1 (2.3)	2 (4.9)	6 (5.1)	1 (2.0)	15 (5.0)	
	Disagree	16 (33.3)	9 (20.5)	13 (31.7)	29 (24.6)	9 (17.6)	76 (25.2)	
	Neutral	15 (31.3)	23 (52.3)	15 (36.6)	49 (41.5)	23 (45.1)	125 (41.4)	
	Agree	11 (22.9)	10 (22.7)	9 (22.0)	30 (25.4)	16 (31.4)	76 (25.2)	
	Strongly agree	1 (2.1)	1 (2.3)	2 (4.9)	4 (3.4)	2 (3.9)	10 (3.3)	
**There were sufficient resources available to promote self-care generally**	Strongly disagree	11 (22.9)	0 (0.0)	3 (7.1)	6 (5.2)	2 (3.9)	22 (7.3)	
	Disagree	16 (33.3)	9 (20.9)	11 (26.2)	31 (26.7)	9 (17.6)	76 (25.3)	
	Neutral	8 (16.7)	17 (39.5)	7 (16.7)	23 (19.8)	16 (31.4)	71 (23.7)	
	Agree	12 (25.0)	14 (32.6)	19 (45.2)	48 (41.4)	19 (37.3)	112 (37.3)	
	Strongly agree	1 (2.1)	3 (7.0)	2 (4.8)	8 (6.9)	5 (9.8)	19 (6.3)	
**There were sufficient resources available to promote self-care for coronavirus**	Strongly disagree	6 (12.8)	3 (6.8)	3 (7.5)	6 (5.1)	3 (5.9)	21 (7.0)	
	Disagree	12 (25.5)	3 (6.8)	7 (17.5)	22 (18.6)	9 (17.6)	53 (17.7)	
	Neutral	9 (19.1)	15 (34.1)	9 (22.5)	19 (16.1)	9 (17.6)	61 (20.3)	
	Agree	18 (38.3)	20 (45.5)	16 (40.0)	60 (50.8)	23 (45.1)	137 (45.7)	
	Strongly agree	2 (4.3)	3 (6.8)	5 (12.5)	11 (9.3)	7 (13.7)	28 (9.3)	
**HCP/PATIENT INTERACTION DYNAMICS & CHANGES AFTER PANDEMIC**								<0.001
**How has your relationship with your service users changed**	Extremely negative	4 (8.5)	0 (0.0)	0 (0.0)	0 (0.0)	1 (2.0)	5 (1.7)	
	Slightly negative	14 (29.8)	6 (13.6)	0 (0.0)	2 (1.7)	1 (2.0)	23 (7.6)	
	No change	7 (14.9)	16 (36.4)	9 (21.4)	19 (16.1)	14 (27.5)	65 (21.5)	
	Slightly positive	18 (38.3)	17 (38.6)	25 (59.5)	63 (53.4)	19 (37.3)	142 (47.0)	
	Extremely positive	4 (8.5)	5 (11.4)	8 (19.0)	34 (28.8)	16 (31.4)	67 (22.2)	
**How has your relationship with other professionals changed**	Extremely negative	0 (0.0)	0 (0.0)	0 (0.0)	2 (1.7)	0 (0.0)	2 (0.7)	
	Slightly negative	9 (19.1)	4 (9.1)	0 (0.0)	11 (9.5)	2 (3.9)	26 (8.7)	
	No change	15 (31.9)	13 (29.5)	11 (26.2)	32 (27.6)	19 (37.3)	90 (30.0)	
	Slightly positive	19 (40.4)	20 (45.5)	24 (57.1)	51 (44.0)	17 (33.3)	131 (43.7)	
	Extremely positive	4 (8.5)	7 (15.9)	7 (16.7)	20 (17.2)	13 (25.4)	51 (17.0)	
**How has access to services changed**	Extremely negative	11 (23.4)	1 (2.3)	0 (0.0)	8 (6.8)	1 (2.0)	21 (7.0)	
	Slightly negative	14 (29.8)	13 (29.5)	6 (14.3)	27 (22.9)	13 (26.5)	73 (24.3)	
	No change	9 (19.1)	11 (25.0)	12 (28.6)	27 (22.9)	10 (20.4)	69 (23.0)	
	Slightly positive	11 (23.4)	15 (34.1)	20 (47.6)	49 (41.5)	17 (34.7)	112 (37.3)	

	Extremely positive	2 (4.3)	4 (9.1)	4 (9.5)	7 (5.9)	8 (16.3)	25 (8.3)	
**How has digital inclusion changed**	Extremely negative	2 (4.3)	2 (4.5)	0 (0.0)	2 (1.7)	1 (2.0)	7 (2.3)	
	Slightly negative	5 (10.6)	6 (13.6)	3 (7.1)	16 (13.8)	6 (12.2)	36 (12.1)	
	No change	8 (17.0)	12 (27.3)	11 (26.2)	35 (30.2)	17 (34.7)	83 (27.9)	
	Slightly positive	28 (59.6)	22 (50.0)	22 (52.4)	53 (45.7)	23 (46.9)	148 (49.7)	
	Extremely positive	4 (8.5)	2 (4.5)	6 (14.3)	10 (8.6)	2 (4.1)	24 (8.1)	
**How has covid changed workforce configuration**	Extremely negative	6 (12.8)	1 (2.3)	0 (0.0)	3 (2.5)	1 (2.0)	11 (3.7)	
	Slightly negative	15 (31.9)	9 (20.9)	2 (4.8)	21 (17.8)	8 (16.3)	55 (18.4)	
	No change	10 (21.3)	14 (32.6)	15 (35.7)	48 (40.7)	23 (46.9)	110 (36.8)	
	Slightly positive	15 (31.9)	16 (37.2)	16 (38.1)	37 (31.4)	13 (26.5)	97 (32.4)	
	Extremely positive	1 (2.1)	3 (7.0)	9 (21.4)	9 (7.6)	4 (8.2)	26 (8.7)	
**How has covid changed training of professionals**	Extremely negative	4 (8.5)	2 (4.8)	0 (0.0)	5 (4.3)	0 (0.0)	11 (3.7)	
	Slightly negative	10 (21.3)	3 (7.1)	1 (2.4)	13 (11.3)	5 (10.2)	32 (10.8)	
	No change	14 (29.8)	9 (21.4)	15 (35.7)	50 (43.5)	22 (44.9)	110 (37.3)	
	Slightly positive	18 (38.3)	23 (54.8)	20 (47.6)	38 (33.0)	19 (38.8)	118 (40.0)	
	Extremely positive	1 (2.1)	5 (11.9)	6 (14.3)	9 (7.8)	3 (6.1)	24 (8.1)	
**How has covid changed the design & cost of commissioned services**	Extremely negative	7 (15.2)	0 (0.0)	0 (0.0)	10 (8.5)	1 (2.0)	18 (6.0)	
	Slightly negative	11 (23.9)	10 (23.3)	6 (14.3)	18 (15.4)	8 (16.0)	53 (17.8)	
	No change	14 (30.4)	13 (30.2)	19 (45.2)	54 (46.2)	23 (46.0)	123 (41.3)	
	Slightly positive	12 (26.1)	16 (37.2)	13 (31.0)	32 (27.4)	16 (32.0)	89 (29.9)	
	Extremely positive	2 (4.3)	4 (9.3)	4 (9.5)	3 (2.6)	2 (4.0)	15 (5.0)	
**LOOKING FORWARD**								0.70
**I am likely to continue to deliver self-care practices which I introduced as a result of the pandemic**	Strongly disagree	0 (0.0)	0 (0.0)	1 (2.4)	1 (0.8)	0 (0.0)	2 (0.7)	
	Disagree	0 (0.0)	0 (0.0)	0 (0.0)	1 (0.8)	1 (2.0)	2 (0.7)	
	Neither agree nor disagree	5 (10.6)	6 (13.6)	2 (4.8)	15 (12.6)	7 (13.7)	35 (11.6)	
	Agree	21 (44.7)	14 (31.8)	20 (47.6)	59 (49.6)	21 (41.2)	135 (44.6)	
	Strongly agree	21 (44.7)	24 (54.5)	19 (45.2)	43 (36.1)	22 (41.1)	129 (42.6)	
**I will continue to actively promote self-care resources**	Strongly disagree	0 (0.0)	0 (0.0)	0 (0.0)	1 (0.8)	0 (0.0)	1 (0.3)	
	Disagree	0 (0.0)	0 (0.0)	0 (0.0)	0 (0.0)	0 (0.0)	0 (0.0)	
	Neither agree nor disagree	1 (2.1)	1 (2.3)	3 (7.1)	1 (0.8)	3 (5.9)	9 (3.0)	
	Agree	16 (34.0)	17 (38.6)	17 (40.5)	59 (49.6)	20 (39.2)	129 (42.6)	
	Strongly agree	30 (63.8)	26 (59.1)	22 (52.4)	58 (48.7)	28 (54.9)	164 (54.1)	
**My patients will be more likely to practice self-care**	Strongly disagree	1 (2.2)	0 (0.0)	0 (0.0)	2 (1.7)	0 (0.0)	3 (1.0)	
	Disagree	9 (19.6)	3 (6.8)	3 (7.1)	7 (5.9)	1 (2.0)	23 (7.6)	
	Neither agree nor disagree	12 (26.1)	17 (38.6)	16 (38.1)	38 (31.9)	17 (33.3)	100 (33.1)	
	Agree	22 (47.8)	19 (43.2)	20 (47.6)	62 (52.1)	27 (42.9)	150 (49.7)	
	Strongly agree	2 (4.3)	5 (11.4)	3 (7.1)	10 (8.4)	6 (11.8)	26 (8.6)	
**My patients will be better equipped & feel more empowered to partake in self-care practices**	Strongly disagree	2 (4.3)	0 (0.0)	0 (0.0)	1 (0.8)	0 (0.0)	3 (1.0)	
	Disagree	9 (19.1)	3 (6.8)	3 (7.1)	10 (8.4)	2 (3.9)	27 (8.9)	
	Neither agree nor disagree	12 (25.5)	17 (38.6)	13 (31.0)	36 (30.3)	19 (37.3)	97 (32.0)	
	Agree	20 (42.6)	19 (43.2)	22 (52.4)	67 (56.3)	21 (41.2)	149 (49.2)	
	Strongly agree	4 (8.5)	5 (11.4)	4 (9.5)	5 (4.2)	9 (17.6)	27 (8.9)	
**There will be a stronger adherence to self-care practices as a first option of care following the pandemic**	Strongly disagree	3 (6.4)	0 (0.0)	0 (0.0)	3 (2.5)	0 (0.0)	6 (2.0)	
	Disagree	12 (25.5)	3 (6.8)	3 (7.1)	9 (7.6)	7 (13.7)	34 (11.3)	
	Neither agree nor disagree	11 (23.4)	14 (31.8)	13 (31.0)	45 (38.1)	15 (29.4)	98 (32.5)	
	Agree	18 (38.3)	23 (52.3)	20 (47.6)	53 (44.9)	21 (41.2)	135 (44.7)	

	Strongly agree	3 (6.4)	4 (9.1)	6 (14.3)	8 (6.8)	8 (15.7)	29 (9.6)	
**The pandemic has made the 'absolute case' for self-care**	Strongly disagree	1 (2.1)	0 (0.0)	0 (0.0)	0 (0.0)	3 (5.9)	4 (1.3)	
	Disagree	8 (17.0)	2 (4.5)	1 (2.4)	10 (8.4)	3 (5.9)	24 (7.9)	
	Neither agree nor disagree	9 (19.1)	8 (18.2)	11 (26.2)	19 (16.0)	11 (21.6)	58 (19.1)	
	Agree	12 (25.5)	20 (45.5)	20 (47.6)	66 (55.5)	20 (39.2)	138 (45.5)	
	Strongly agree	17 (36.2)	14 (31.8)	10 (23.8)	24 (20.2)	14 (27.5)	79 (26.1)	
**I will personally be more likely to practice self-care**	Strongly disagree	0 (0.0)	0 (0.0)	0 (0.0)	0 (0.0)	0 (0.0)	0 (0.0)	
	Disagree	3 (6.4)	0 (0.0)	0 (0.0)	2 (1.7)	1 (2.0)	6 (2.0)	
	Neither agree nor disagree	18 (38.3)	8 (18.6)	8 (20.0)	27 (22.7)	8 (15.7)	69 (23.0)	
	Agree	14 (29.8)	20 (46.5)	16 (40.0)	49 (41.2)	29 (56.9)	128 (42.7)	
	Strongly agree	12 (25.5)	15 (34.9)	16 (40.0)	41 (34.5)	13 (25.5)	97 (32.3)	
		**Doctor**	**Nurse**	**Service**	**Pharmacy**	**SP/Other**	**Total**	**p-value**
**USE OF TECHNOLOGY DURINFG THE PANDEMIC**								0.26
Email	Reduced significantly	0 (0.0)	0 (0.0)	1 (2.4)	1 (0.9)	0 (0.0)	2 (0.7)	
	Reduced slightly	2 (4.5)	2 (4.5)	1 (2.4)	0 (0.0)	0 (0.0)	3 (1.0)	
	No change	13 (29.5)	13 (29.5)	10 (24.4)	40 (34.5)	13 (26.0)	85 (28.4)	
	Increased slightly	19 (43.2)	19 (43.2)	16 (39.0)	43 (37.1)	18 (36.0)	121 (40.5)	
	Increased significantly	10 (22.7)	10 (22.7)	13 (31.7)	32 (27.6)	19 (38.0)	88 (29.4)	
	**Total**	48 (100.0)	44 (100.0)	41 (100.0)	116 (100.0)	50 (100.0)	299 (100.0)	
Text Messaging (SMS)	Reduced significantly	0 (0.0)	0 (0.0)	0 (0.0)	0 (0.0)	0 (0.0)	0 (0.0)	
	Reduced slightly	0 (0.0)	1 (2.4)	0 (0.0)	1 (0.9)	0 (0.0)	2 (0.7)	
	No change	3 (6.3)	15 (35.7)	20 (51.3)	34 (29.3)	17 (35.4)	89 (30.4)	
	Increased slightly	11 (22.9)	14 (33.3)	11 (28.2)	46 (39.7)	17 (35.4)	99 (33.8)	
	Increased significantly	34 (70.8)	12 (28.6)	8 (20.5)	35 (30.2)	14 (29.2)	103 (35.2)	
	**Total**	48 (100.0)	42 (100.0)	39 (100.0)	116 (100.0)	48 (100.0)	293 (100.0)	
Telephone	Increased significantly	0 (0.0)	0 (0.0)	0 (0.0)	0 (0.0)	1 (2.0)	1 (0.3)	
	Increased slightly	0 (0.0)	1 (2.3)	0 (0.0)	0 (0.0)	0 (0.0)	1 (0.3)	
	No change	7 (14.6)	7 (15.9)	2 (5.0)	39 (33.6)	6 (12.2)	61 (20.5)	
	Reduced slightly	21 (43.8)	13 (29.5)	11 (27.5)	29 (25.0)	19 (38.8)	93 (31.3)	
	Reduced significantly	20 (41.7)	23 (52.3)	27 (67.5)	48 (41.4)	23 (46.9)	141 (47.5)	
	**Total**	48 (100.0)	44 (100.0)	40 (100.0)	116 (100.0)	49 (100.0)	297 (100.0)	
Recommending Online Resources	Reduced significantly	0 (0.0)	1 (2.3)	1 (2.4)	1 (0.9)	0 (0.0)	3 (1.0)	
	Reduced slightly	0 (0.0)	2 (4.7)	2 (4.9)	3 (2.6)	0 (0.0)	7 (2.4)	
	No change	4 (8.5)	6 (14.0)	6 (14.6)	8 (6.8)	7 (14.3)	31 (10.4)	
	Increased slightly	8 (17.0)	13 (30.2)	13 (31.7)	34 (29.1)	15 (30.6)	83 (27.9)	
	Increased significantly	35 (74.5)	21 (48.8)	19 (46.3)	71 (60.7)	27 (55.1)	173 (58.2)	
	**Total**	47 (100.0)	43 (100.0)	41 (100.0)	117 (100.0)	49 (100.0)	297 (100.0)	
Home Monitoring	Reduced significantly	0 (0.0)	0 (0.0)	0 (0.0)	0 (0.0)	0 (0.0)	0 (0.0)	
	Reduced slightly	0 (0.0)	0 (0.0)	1 (2.6)	0 (0.0)	0 (0.0)	1 (0.3)	
	No change	4 (8.3)	17 (39.5)	24 (63.2)	26 (22.4)	26 (57.8)	97 (33.4)	
	Increased slightly	21 (43.8)	11 (25.6)	8 (21.1)	51 (44.0)	11 (24.4)	102 (35.2)	
	Increased significantly	23 (47.9)	15 (34.9)	5 (13.2)	39 (33.6)	8 (17.8)	90 (31.0)	
	**Total**	48 (100.0)	43 (100.0)	38 (100.0)	116 (100.0)	45 (100.0)	290 (100.0)	
Remote Monitoring by the Care Team	Reduced significantly	1 (2.1)	1 (2.4)	0 (0.0)	1 (0.9)	0 (0.0)	3 (1.0)	
	Reduced slightly	1 (2.1)	1 (2.4)	1 (2.6)	2 (1.7)	3 (6.5)	8 (2.7)	

	No change	11 (22.9)	13 (31.0)	13 (33.3)	42 (36.2)	24 (52.2)	103 (35.4)	
	Increased slightly	21 (43.8)	16 (38.1)	15 (38.5)	40 (34.5)	10 (21.7)	102 (35.1)	
	Increased significantly	14 (29.2)	11 (26.2)	10 (25.6)	31 (26.7)	9 (19.6)	75 (25.8)	
	**Total**	48 (100.0)	42 (100.0)	39 (100.0)	116 (100.0)	46 (100.0)	291 (100.0)	
Use of Smartphone Apps	Reduced significantly	0 (0.0)	0 (0.0)	0 (0.0)	0 (0.0)	0 (0.0)	0 (0.0)	
	Reduced slightly	0 (0.0)	1 (2.3)	0 (0.0)	0 (0.0)	1 (2.1)	2 (0.7)	
	No change	16 (33.3)	11 (25.6)	10 (26.3)	20 (17.9)	7 (14.6)	64 (22.1)	
	Increased slightly	19 (39.6)	20 (46.5)	18 (47.4)	51 (45.5)	25 (52.1)	133 (46.0)	
	Increased significantly	13 (27.1)	11 (25.6)	10 (26.3)	41 (36.6)	15 (31.3)	90 (31.1)	
	**Total**	48 (100.0)	43 (100.0)	38 (100.0)	112 (100.0)	48 (100.0)	289 (100.0)	
