# Supplementary material for: How has COVID-19 changed healthcare professionals’ attitudes to self-care? A mixed methods research study
Source: PLoS One. 2023 Jul 24;18(7):e0289067. doi: 10.1371/journal.pone.0289067 (PMC10365300; doi:10.1371/journal.pone.0289067)
Supplement: S2 Table — (DOCX) [file pone.0289067.s002.docx]

S2 Table CAPPS Overarching themes

| **Categories** | **Subcategories** | **Thematic framework** |
| --- | --- | --- |
| **Patient/Client Attitudes to Self-care** | - Barriers | 1. Dependency 2. Medicalisation 3. Limited accessibility 4. Cultural 5. Lack of resources 6. Limited health literacy & access to information 7. Physical constraints 8. Time 9. Insufficient support |
|  | - Responsibility | 1. Professional 2. Patients/Public 3. Boundaries of self -care |
| **Professional Approaches to Self-care** | - Scope of Self-care | 1. Patient self-management of conditions 2. Wellbeing 3. Supported self-management 4. Lifestyle & fitness 5. Better informed patients 6. Beyond the medical theme |
|  | - Motivation | 1. General health 2. Self-management |
|  | - Attitudes | 1. More open to self-care following Covid-19 2. Extended professional support for self-care 3. Requires quality of information & advice 4. Boundaries of professional/self-care 5. Personal self-care |
| **COVID-19 Specific** | - Drivers | 1. Self-care recommendations 2. Change of attitude in professionals & patients |
|  | - Role of technology - Barriers | 1. Self-monitoring 2. Online information 3. Feeling of alienation/abandonment in patients 4. Unmet needs 5. Digital inclusion/exclusion |
| **Future** | - Changes & Potential | 1. Shared acceptance of principle of self-care 2. Use of online advice 3. Information 4. Change in professional role 5. Change in public approach 6. Self-monitoring |
|  | - Risks & limitations | 1. Unmet needs 2. Alienation 3. Access 4. Quality of information 5. New relationship 6. Training need 7. Uncertain durability of Covid-19 changes 8. Requires revised/additional medical training |
