# Supplementary material for: How has COVID-19 changed healthcare professionals’ attitudes to self-care? A mixed methods research study
Source: PLoS One. 2023 Jul 24;18(7):e0289067. doi: 10.1371/journal.pone.0289067 (PMC10365300; doi:10.1371/journal.pone.0289067)
Supplement: S1 File — (PDF) [file pone.0289067.s003.pdf]

Changes in the attitudes and practice of health & social care professionals with respect to self-care as a result of the Covid-19 pandemic (CAPPS)

eSurvey (on Qualtrics)

SEE LINK:
Section 1: Survey Questions: https://imperial.eu.qualtrics.com/jfe/form/SV_5vynl3kRrEfgEJ0

Q1
COVID-19 has fundamentally changed the way health & social care services are delivered at a time where self-care has also revealed itself as the critical answer to tackling the pandemic. Self-care praxis remains largely understudied, and in particular how the attitudes of health & social care staff may have changed with respect to embedding good health-seeking self-care behaviours in the clients they serve in the new setting.

Imperial College London Self-Care Academic Unit (SCARU) is conducting a qualitative research study to explore this further. Please take a moment to review the Participant Information Sheet of this study which also has ethical approvals in place.

Thank you for taking part in this 5-10 min eSurvey. Please feel free to disseminate study information & link to your personal contacts of health & social care professionals.

Q2 Please confirm that you consent to participate in this survey.

- ○ Yes (1)
- ○ No (2)

Q3
This section covers the advice & support you give to those you help in your professional role (such as patients, clients & individuals receiving health & social care)
Which aspects of self-care did you actively encourage prior to the pandemic?

▢ Improving general wellbeing (1)

▢ Making healthy lifestyle choices (e.g. exercise or diet) (2)

▢ Building self-care capacity as part of routine professional care (8)

▢ Preventing non-communicable diseases (e.g. type 2 diabetes or obesity) (3)

▢ Preventing infectious diseases (4)

▢ Managing Infectious diseases (14)

▢ Improving mental health (5)

▢ Preventing mental illness (15)

▢ Managing existing mental illness (6)

▢ Managing minor illness & common conditions (e.g. headache or the common cold) (7)

▢ Managing chronic long-term conditions (9)

▢ Managing cancer care (10)

▢ Managing prescribing, dispensing & encouraging appropriate use of medicines (11)

▢ None of the above (12)

▢ Other (please specify) (13) ____

Q4 **Perceived importance of self-care to patients before the pandemic?**

- ○ Very unimportant (1)
- ○ Unimportant (2)
- ○ Neutral (3)
- ○ Important (4)
- ○ Very important (5)

Q5 Perceived capacity and empowerment of patients to self-care before the pandemic?

- ○ 0 (0)
- ○ 1 (1)
- ○ 2 (2)
- ○ 3 (3)
- ○ 4 (4)
- ○ 5 (5)
- ○ 6 (6)
- ○ 7 (7)
- ○ 8 (8)
- ○ 9 (9)
- ○ 10 (10)

Q6 Prior to the pandemic, what were YOUR main motivations to support people to self-care in your professional role? (Select one or more)

- ▢ To promote the rational use of products & services (8)
- ▢ To help reduce pressure on scarce NHS resources (6)
- ▢ To help me reduce my workload (3)
- ▢ To promote health literacy (9)
- ▢ It is more convenient to the client/user (4)
- ▢ It is empowering to the client/user (10)
- ▢ To help individuals prevent certain conditions or minimise likelihood of exacerbations (2)
- ▢ The option to self-care for some conditions is the superior mode of care (1)
- ▢ Other (please specify) (5) ____

Q7 **This subsection covers your own attitudes, perception & practices in relation to self-care in your own personal life BEFORE the pandemic.**

Before the advent of COVID-19, how strongly did you feel that self-care was important to YOU personally in your daily life?

- ○ Very unimportant (1)
- ○ Slightly unimportant (2)
- ○ Neutral (3)
- ○ Slightly important (4)
- ○ Very important (5)

Q8 Which of these aspects of self-care did **you** actively pursue prior to the pandemic? (Select one or more)

- ☐ Improving general wellbeing (1)
- ☐ Making healthy lifestyle choices (e.g. exercise or diet) (2)
- ☐ Building self-care capacity as part of routine professional care (8)
- ☐ Preventing non-communicable diseases (e.g. type 2 diabetes or obesity) (3)
- ☐ Preventing infectious diseases (4)
- ☐ Managing Infectious diseases (14)
- ☐ Improving mental health (5)
- ☐ Preventing mental illness (15)
- ☐ Managing existing mental illness (6)
- ☐ Managing minor illness & common conditions (e.g. headache or the common cold) (7)
- ☐ Managing chronic long-term conditions (9)
- ☐ Managing cancer care (10)
- ☐ Managing prescribing, dispensing & encouraging appropriate use of medicines (11)
- ☐ None of the above (12)
- ☐ Other (please specify) (13) ____

Q9
This section covers your daily work & the advice you give now (since the advent of the COVID-19 pandemic) to those individuals you look after in your professional role.

Are you more or less likely to recommend & support your service users to self-care in your daily work as a result of the pandemic?

- Significantly less likely (0)
- Slightly less likely (1)
- No change (2)
- Slightly more likely (3)
- Significantly more likely (4)

Q10 Since the advent of the pandemic, which of these broad aspects are YOU more likely to encourage & support people to achieve? (Select one or more)

- ☐ Improving general wellbeing (1)
- ☐ Making healthy lifestyle choices (e.g. exercise or diet) (2)
- ☐ Building self-care capacity as part of routine professional care (8)
- ☐ Preventing non-communicable diseases (e.g. type 2 diabetes or obesity) (3)
- ☐ Preventing infectious diseases (4)
- ☐ Managing Infectious diseases (14)
- ☐ Improving mental health (5)
- ☐ Preventing mental illness (15)
- ☐ Managing existing mental illness (6)
- ☐ Managing minor illness & common conditions (e.g. headache or the common cold) (7)
- ☐ Managing chronic long-term conditions (9)
- ☐ Managing cancer care (10)
- ☐ Managing prescribing, dispensing & encouraging appropriate use of medicines (11)
- ☐ None of the above (12)
- ☐ Other (please specify) (13) ____

Q11 Main HCP motivations to support people to self-care since the advent of COVID-19 pandemic?

☐ To promote of the rational use of products & services (8)

☐ It can help reduce pressure on scarce NHS resources (6)

☐ To help me reduce my workload (3)

☐ To promote of health literacy (9)

☐ It is more convenient to the client/user (4)

☐ It is empowering to the client/user (10)

☐ To help individuals prevent certain conditions or minimise likelihood of exacerbations (2)

☐ The option to self-care for some conditions is the superior mode of care (1)

☐ Other (please specify) (5)

____

Q12 Perceived importance of self-care to patients since the advent of COVID-19 pandemic?

○ Extremely unimportant (1)

○ Slightly unimportant (2)

○ Neutral (3)

○ Slightly important (4)

○ Extremely important (5)

Q13 Perceived capacity/empowerment of service users to self-care since the advent of the pandemic (where 0 is not at all & 10 is extremely)

- ○ 0 (0)
- ○ 1 (1)
- ○ 2 (2)
- ○ 3 (3)
- ○ 4 (4)
- ○ 5 (5)
- ○ 6 (6)
- ○ 7 (7)
- ○ 8 (8)
- ○ 9 (9)
- ○ 10 (10)

Q14 This section covers YOUR PERSONAL APPROACH to self-care during the pandemic.

Which aspect of self-care did you personally pursue during the pandemic? (Select one or more)

- ☐ Improving general wellbeing (1)
- ☐ Making healthy lifestyle choices (e.g. exercise or diet) (2)
- ☐ Building self-care capacity as part of routine professional care (8)
- ☐ Preventing non-communicable diseases (e.g. type 2 diabetes or obesity) (3)
- ☐ Preventing infectious diseases (4)
- ☐ Managing Infectious diseases (14)
- ☐ Improving mental health (5)
- ☐ Preventing mental illness (15)
- ☐ Managing existing mental illness (6)
- ☐ Managing minor illness & common conditions (e.g. headache or the common cold) (7)
- ☐ Managing chronic long-term conditions (9)
- ☐ Managing cancer care (10)
- ☐ Managing prescribing, dispensing & encouraging appropriate use of medicines (11)
- ☐ None of the above (12)
- ☐ Other (please specify) (13) ____

Q15
The following subsection covers your views on the use of resources & technology during the pandemic.

To what extent do you agree or disagree with the following statements.

	Strongly disagree (1)	Disagree (2)	Neutral (3)	Agree (4)	Strongly agree (5)
I have the resources & proficiency to promote self-care in my practice (9)	○	○	○	○	○
My clients/patients are adequately prepared/trained to self-care (10)	○	○	○	○	○
My clients/patients have the competency & resources to self-care (11)	○	○	○	○	○
There were sufficient resources available to promote self-care generally (4)	○	○	○	○	○
There were sufficient resources available to promote self-care for coronavirus (6)	○	○	○	○	○

Display This Question:

If The following subsection covers your views on the use of resources & technology during the pandem... = There were sufficient resources available to promote self-care generally [Strongly disagree]

And The following subsection covers your views on the use of resources & technology during the pandem... = There were sufficient resources available to promote self-care generally [Disagree]

And The following subsection covers your views on the use of resources & technology during the pandem... = There were sufficient resources available to promote self-care for coronavirus [Strongly disagree]

And The following subsection covers your views on the use of resources & technology during the pandem... = There were sufficient resources available to promote self-care for coronavirus [Disagree]

Q16
If you felt that resources to self-care were insufficient, which of the following experiences did you pursue in search of appropriate resources (information/website/literature) to support self-care? (Select one or more)

- ▢ I created my own self-care sources for dissemination (3)
- ▢ I commissioned the development of new resources (5)
- ▢ I located resources I had not used before (e.g. new guidelines, online forums) (6)
- ▢ I adapted existing resources (7)
- ▢ None of the above (9)
- ▢ Other (please specify) (12) ____

Page Break

Q17

To what extent do you feel the pandemic has influenced usage of the following technologies to support your patients/clients to self-care?

	Reduced significantly (1)	Reduced slightly (2)	No change (3)	Increased slightly (4)	Increased significantly (5)
Email (1)	○	○	○	○	○
Text (SMS) messaging (2)	○	○	○	○	○
Video conference (3)	○	○	○	○	○
Telephone (4)	○	○	○	○	○
Recommending online resources (5)	○	○	○	○	○
Home monitoring (e.g. glucometer, blood pressure monitor etc.,) (8)	○	○	○	○	○
Remote monitoring by the care team (10)	○	○	○	○	○
Use of smartphone apps (9)	○	○	○	○	○
Other (please specify) (11)	○	○	○	○	○

Q18 Perceived general barriers to self-care? (Select one or more)

- ☐ Lack of evidence of effectiveness of self-care interventions (2)
- ☐ Lack of appropriate information to share with people (3)
- ☐ Difficulties in access to services & professionals (4)
- ☐ Digital exclusion (5)
- ☐ Barriers caused by new digital or telephone interactions (6)
- ☐ Lack of funding (7)
- ☐ Health inequalities (8)
- ☐ Transition of services from face-to-face to other formats (9)
- ☐ Patient/client understanding of self-care (12)
- ☐ Language barriers (13)
- ☐ Individual reluctance to engage or take responsibility (14)
- ☐ Health literacy (15)
- ☐ Dependency on professional role (16)
- ☐ Communication barriers due to hearing difficulties (17)
- ☐ Lack of equipment/hardware with which to communicate (18)
- ☐ Insufficient IT skills (19)
- ☐ Professional understanding of self-care (21)
- ☐ Time constraints (22)

- ☐ Professional resistance to sharing responsibility (23)
- ☐ Lack of IT training (24)
- ☐ Lack of consistency in approach or messages amongst professionals (25)
- ☐ Interprofessional communication (26)
- ☐ Professional understanding of alternatives to face-to-face interactions (27)
- ☐ Other (please specify) (29) ____

Q19

This section covers your practice & attitudes to self-care AFTER the COVID-19 pandemic (i.e. when vaccines become widely available & lockdowns are no longer necessary).

Do you think there will be a positive or negative effect in the following as a result of the changes in self-care attitudes & practices after the pandemic?

	Extremely negative (1)	Slightly negative (2)	No change (3)	Slightly positive (4)	Extremely positive (5)
Your relationship with your patients/clients (1)	○	○	○	○	○
Your relationship with other professionals (2)	○	○	○	○	○
Access to services (3)	○	○	○	○	○
Digital inclusion (4)	○	○	○	○	○
Workforce configuration (5)	○	○	○	○	○
Training of professionals (6)	○	○	○	○	○
Design & cost of commissioned services (7)	○	○	○	○	○

Q20 Please state the extent to which you agree or disagree with the following statements regarding adherence to self-care practices AFTER the pandemic.

	Strongly disagree (1)	Disagree (5)	Neither agree nor disagree (2)	Agree (3)	Strongly agree (4)
I am likely to continue to deliver self-care practices which I introduced as a result of the pandemic (1)	○	○	○	○	○
I will continue to actively promote self-care resources (2)	○	○	○	○	○
My clients/patients will be more likely to practice self-care (3)	○	○	○	○	○
My clients/patients will be better equipped & feel more empowered to partake in self-care practices (4)	○	○	○	○	○
There will be a stronger adherence to self-care practices as a first option of care following the pandemic (6)	○	○	○	○	○
The pandemic has made the 'absolute case' for self-care	○	○	○	○	○

(7) I will personally be more likely to practice self-care (5)	○	○	○	○	○

Q21
This section deals with demographics
What is your gender?

○ Male (1)

○ Female (2)

○ Other (please specify) (3) ____

Q22 How old are you? (In years)

____

Q23 What is your occupation / designation?

____

Q24 What is the first part of your postcode? (E.g. if your postcode is 'SW18 5FP', please type 'SW18')

____

Q25 Which best describes your field of work?

- ○ Primary care (1)
- ○ Secondary care (2)
- ○ Public health (3)
- ○ Social care (4)
- ○ Voluntary sector (5)
- ○ Other (please specify) (6) ____

Q26 What is your ethnicity?

- ○ White (1)
- ○ Mixed/multiple ethnic groups (2)
- ○ Asian/Asian British (3)
- ○ Black/African/Caribbean/Black British (4)
- ○ Other (please specify): (5) ____

Q27 Thank you for taking the time to answer our survey.

Researchers from Imperial College London are looking to interview up to 30 participants (via telephone, Skype or Microsoft Teams) to learn more about specific themes. Interviews will last 25-35 minutes. Please provide your name & contact details (below) if this interests you. We are happy to answer any questions on the study & can fix a suitable time and date for an interview. Thank you.

- ○ Name: (1) ____
- ○ E-mail: (2) ____
- ○ Mobile number: (3) ____

End of Block: Block 1
