# Supplementary material for: How has COVID-19 changed healthcare professionals’ attitudes to self-care? A mixed methods research study
Source: PLoS One. 2023 Jul 24;18(7):e0289067. doi: 10.1371/journal.pone.0289067 (PMC10365300; doi:10.1371/journal.pone.0289067)
Supplement: S2 File — (PDF) [file pone.0289067.s004.pdf]

CAPPS

Professional Attitudes to Self Care

Categories and Themes v1.0

A Scope of self care

	Category	Themes	Comments
**A**	**Scope of Self care**	**Patient**<br><br>**Management of conditions**	Patients<br>Things they can do to make their conditions better for example taking hayfever medication if their asthma and changing this soap if they have eczema for example<br><br>GP – self-care is what patients do to help themselves before contacting you<br><br>For example headache using apps and websites discussing with friends and then consulted at an appropriate time<br><br>Also comes up with a pharmacist nurse other professionals GPs
		**Wellbeing**	Staff recognising that without us patients were grouped after we need to look after her own well-being recognising that our lives outside of an impact now work itself<br><br>Self making sure you have time for yourself taking steps to manage and get help when there are problems<br><br>Self-care is making time to yourself, both in terms of physical and mental health<br><br>Taking steps to look after well-being
		**Supported**<br><br>**Self management**	**it is also about buying into the physiotherapy side of things and doing the exercises are given to maximise your chance of recovery.**

		**Lifestyle & Fitness**	Personally diet and exercise are becoming extremely important though following the advice given was always easy<br><br>**This extends to having lifestyle it is healthy and staying away from people of Covid!**<br><br>The him and for others it is about maintaining fitness in the current environment<br>Internal environment adapting to the new environment<br>External particularly with Covid coping with political financial and social changes<br>**Self-care is looking after themselves using the resources available**<br>**People looking after themselves**<br>**mindfully healthy habits exercise mindfulness**<br>Lifestyle, diet and exercise<br><br>Important things about people considering what's important increasingly<br>deal with what people themselves who is important people have been interested in hobbies such as gardening what they like doing doing something that interests them such as going out and mindfulness and give the tools to help them<br><br>Personally my mind and health are important particularly generous reflective walk when mindfulness is practice<br><br>Headspace and calm apps prove very useful with meditation and music
		**Informed**	Keeping up-to-date with self-care meant using trusted medicines using own knowledge but also keeping up-to-date with the latest papers and research did not that easy to get<br><br>**Self-care means this will be informed**<br>**Self-care is a cultural thing ethical dimension particular endearing dealing with neurodevelopmental disorders which may require the carer to be the self carer**<br><br>customers can be stressed and worried increasing need for signposting and advice<br>Giving tailored advice with long-term conditions what is achievable and practical part of a partnership decision
		**Beyond the medical**	**The medical model has its limitations this is not always recognised by doctors when dealing with self-care**<br>**In the absence of other care alternative medicine has been useful in combining complementary medicine with allopathic medicine**

B Barriers

	Category	Theme	
		**Dependency**	? Dependency<br>Personal resilience appears to have gone as has family wisdom.<br><br>In her BAM is the culture is of respect and dependent on GP e population there is not as much recourse to self-care before contacting<br><br>It is a GP dependent area and not happy when seeing other professionals<br><br>the practice has alternative care practitioners including pharmacists and physios but patients are not happy generally when asked to see another practitioner<br><br>is overlapped in common rare services we are also always seeking the help of the medical profession, creating a need and a dependency<br><br>Symptom checkers have been used theoretically this has improved access of UC consultant accurate kicks but all of these again have facilitated the dependence on our an answer from a general practitioner which is not necessary
		**Medicalisation**	Feeling that the non-medical side of self-care has become increasingly medicalised and shifted to a professional arena.<br><br>Paragraph docs perhaps part of the problem of over medicalisation. For example, cough being considered as cancer after three weeks and marginal vitamin D levels being considered as an illness requiring testing and medicalisation with definitions based on reference ranges which result in significant diagnosis<br><br>Paternalism is a problem

			Although there is availability of Internet information to promote for centre GP<br><br>Particularly in the pandemic – expose the problem as the need unnecessary clinical reassurance arose needing to be seen is not necessary and neither was examination investigation in the people are used to this<br><br>90% of diagnosis are made on history and yet suggest that the patient needs to be examined in this very
		**Access**	A particular problem has been that it is a green hospital sincere days of Covid had no visitors and now still only two visitors a week<br><br>In the case of referral the difficulties of been that although lumps and bumps are referable there are a hundred different types of sarcoma and is impossible to provide many early warning signs for people to watch out for part from night pain<br><br>access to translators is a problem even when booking an appointment<br><br>The elderly in particular the difficulties with technology have been a problem
		**Information**	There is a need to give people the confidence to self-care along with necessary support otherwise is a danger that people will feel abandoned rather than empowered.<br><br>Digital inclusiveness has been an issue and therefore they have had to reduce their dependence on remote access – anything elderly did not have access to the NHS at and culturally self-care is not a priority<br><br>More recently don’t come if not vaccinated has left people to rely on Internet which is not always the most effective<br><br>Don’t think that information can provide enough for everyone enough
		**Cultural**	It is impossible to get through to people particularly women were walked prayers as part of the exercise in understanding family dynamic a good are doing it does mean the elderly patient parents of left devices

			The Bame population in particular remains motivated to get antibiotics for every ill, but consistently passages with practice will decide whether any campaign to reduce unnecessary antibiotic to successful<br><br>The particular ethnic group does not like to have smears and very rarely used ill so there are fewer opportunities for opportunistic intervention<br><br>**Lifestyle advice has been more difficult with the older population who often need personal health**<br><br>Culture<br>the younger often have knowledge and respond particularly well to signposting<br>elderly often prefer to be directed told what to do<br>without understanding conditions with no wish to do so there is the feeling that 'is the doctoral make it better' is<br><br>**Unfortunately access to treatment is too simple many people want medicine because that's the way they expect to be treated**<br>Culture is to seek from a doctor
		**Resources**	**In the early stages was a complete knowledge vacuum and PPE just was not available despite what said publicly and she had to source scrubs**<br><br>The elderly Bame population as a particular problem because they find it difficult to access resources
		**Health literacy**	there needs to be a balance to ensure people are not barred – health literacy is vital but we can't assume it is universal<br><br>**People are unable to understand and follow instructions such as having a good diet**
		**Physical**	some patients are physically incapable of looking after themselves and require such as the people with restricted mobility

		**Time**	The reduction in the time for phone calls is not an effective use of the doctors time<br><br>Practice practising in an under doctored area but having 15 minute consultations which is the minimum required<br>Time is a big barrier<br>priorities<br>the pandemic has been a huge issue because people could not go out and therefore it impacted negatively
		**Support**	**Is often family members who are responsible for the self-care of elderly people. Most people do have an element of support its quest finding it and providing appropriate advice**

**C**	**Responsibility**	Professional	SC has been everybody's job but nobody's responsibility<br><br>COVID has made the need & interest in SC universal.<br><br>GP has turned to it in a major way (online consultations) but particularly telephone consultations.<br><br>. It's more considered to be a hobby for some people. When roles are developed in ICS, SC is unlikely to feature in their strategic documents because they won't be judged on it.<br><br>More focus on personalized medicine & integrated care again a very pater<br><br>If HCP are not allowed to me with patients, then they will be advocating and emphasizing self-care, but there is a limit to how much self-care can accomplish particularly for patients who have clinical needs and entered a pathway.<br><br>Professional's- it is not standard in routine GP to discuss self-care options. More reliance on OTC/POM or investigations. This is what most patients see a doctor.

		People	Person's POV- there is no barrier as they are doing it already. The barrier to better self-care is access to evidence-based advice & recommendations.
		Universal & Boundaries	COVID has made the need & interest in SC universal.<br><br>Previously SC was driven by a different silos of SC stakeholders, but there are now many more SC enthusiasts<br><br>There is no one-size-fit al<br><br>We may be a long way before HCP recommend self-car as the first go-to option, but this will change especially since CIVD- where NHS recommendations were self-care first.
**D**	**Different perspectives / attitudes**		
**E**	**Limits of self-care**		
	**Self-care policy**		

F	COVID	**Self care recommendations**	Avoiding NHS sites & NHS professionals due to social distancing emphasizes the importance of SC<br><br>111 covid clinical assessment service- was all under the section of self-care e(this was unprecedented).<br><br>All advice that government gave (face, space, masks) was all about self-care. And the fin<br>Giving people viable self-care options is crucial and we're seeing that. No there are far more viable options
		**Access**	there needs to be a balance to ensure people are not barred – health literacy is vital but we can't assume it is universal<br><br>[illegible]<br>A further problem has been the turn Covid patients tend to present data with more extensive disease because they have been missed is not want to bother professionals with a perception a GP surgeries are closed<br><br>Particularly this to be notice with no referrals come from physios where this was a very common referral route free Covid<br><br>During the first wave many people fell off the radar completely may did respect lockdown but this meant they did not ring at all for appointments numbers of GPs are cocky to be is the time available has been cut significantly even though telephone consultations did not result in short consultations
		**Unmet need**	This will always be a balance<br><br>We are now seeing particularly a rebound in the middle zone of the continuum with a massive demand from people who have been by default self caring and storing problems rather than perhaps dealing with them<br><br>The family this means it is more difficult and to get support and they are unable to see the progress the family member before the return home is also more difficult the hospital to monitor progress<br><br>In the case of referral the difficulties of been that although lumps and bumps are referable there are a hundred different types of sarcoma and is impossible to provide many early warning signs for people to watch out for part from night pain

			Clinical nurse specialist have noted people of usually been seen several times before they are admitted free Covid now they will only seen one or two people previously they would have had one or two at the MDT meeting but now have up to 6 because of delays in diagnosis<br><br>They are now finding increasingly that malignancies featuring will avoid contact during lockdown<br><br>Mental health issues have a particular stigma in the Bame population though they suffered particularly with anxiety symptoms talking therapies include effective once you can be engaged<br><br>Young people particularly struggled children's anxiety has been a problem with the impossible to signpost to a good local system for talking therapies

G Technology

G	**Technology**	**Self Monitoring**	Accelerated roll out of remote monitoring, BP, Oximetry<br><br>**measurement**<br><br>Amongst people and professionals there has been a significant move towards acceptance of home monitoring including oximeters and BP machines.<br><br>the ability the availability of the kit itself has also been a significant change having been discussed previously<br><br>Getting patients to buy their own machines for monitoring blood pressure giving machines for diabetic monitoring has demonstrated a great willingness for people to do their own monitoring local schemes to give IPP machines have been very effective

			Else oximetry has also been a means for patient empowerment and is used as an opportunity to ensure self-care messages are heard<br><br>People are now used to monitoring their own health including blood pressure and oximetry and diabetes and this is like to be a permanent change<br>Self-care fundamentally changing the people put their own thermometers pulse oximeters for heart rate as well as oximetry BP machines and even the simple things such as wife's and hand sanitiser are now part of the mainstay of self-care<br><br>Lateral flow tests in huge success though advice to pharmacist
		Online information	change of focus not all positive<br><br>online diagnosis<br><br>encouragement of self-care-Google since pandemic<br><br>Many digital health platforms are now being used which may be of questionable benefit to individuals compared with professional input.<br><br>Many of the platforms are driven by commerce and may well be money dressed up as good for everyone
		Alienation/distance	Many professionals have missed the personal contacts<br><br>The rise in telephone triage may well have led to this dissatisfaction on both sides because the loss of the soft cues the empathy and the consideration of belief which may be lost in online consultations<br><br>During Covid 19 with the move away from face-to-face consultations telephone consultations with people who may not know is been a process of alienation between the clinician and patient<br><br>with practitioner health two thirds of people he never meet but it is possible using soon to mitigate the lack of personal contact in the consultation and develop a level of trust.<br><br>This is not the same as the triaging process with the ethos of the value of the consultation may well be lost unfortunately there are no cough points for conducting valuable consultation

		Positive changes	The ability to distribute test results has been a godsend however is not clear whether perhaps technology can be a hindrance. This is particularly been the case the symptom checkers. Its role has yet to be clarified and too often AI doesn't make a decision at all that it still requires human decision the discussion already altered by the AI process.<br><br>However technology can be very convenient and can direct people to good sources where these are known<br><br>Hospital moved into new build hospital in 2018 bedside patient screens with free Wi-Fi which have made a difference most people are now able to talk to relatives via video links.<br><br>Video calls of saved a lot of time and effort in that even within the hospital there is no running around to meetings too much time has been saved.<br><br>Increasingly mobile phones are being used<br><br>90% of diagnosis are made on history and yet suggest that the patient needs to be examined in this very<br><br>We moved to phone calls including an electronic message book of patients to access patient records access is up to 50% and it fantastic of patients can look into their own full record of this has been an important thing electronic message book with an unstructured message has also been a new development which has proved valuable<br>E web access has been a double-edged sword in the GPs are often dealing up to hundred contacts a day and they are effectively becoming Dr Google as People's behaviour changes to googling their own GP<br><br>CP CS services means that people are referred now to pharmacist for minor illness and is important to have a route for dealing with these in a red flag is identified<br><br>Technology has not changed much for pharmacists as most of it has been face-to-face however an important part of the bowl has been helping people set up the NHS at order medication and to keep track of their information
		Digital inclusion	Digital inclusiveness has been an issue and therefore they have had to reduce their dependence on remote access – anything elderly did not have access to the NHS at and culturally self-care is not a priority

			Diabetes has been a particular area which has been possible to have joint meetings including daughters and sons in law<br><br>Technology has played an increasing part are perhaps less for the Bame population in Slough. They have had to continued with a much higher percentage of face-to-face consultations in order to make sure people are not missed.<br><br>They've had to turn to increasingly to mobile phone use though the elderly often do not have access to the technology and do not understand it<br><br>The elderly in particular the difficulties with technology have been a problem<br>followed up by the pharmacists with check telephone calls regularly<br>lots of apps for people who can easily access IT including headspace and calm apps<br>CPCS It was felt this could not be left up to the patient to contact Dr and directory are is essential
		Negative issues	Although they have moved to video consultations now is take much longer than five minutes and difficulties with connection in such has reduced the benefit of these calls<br><br>However the technology when it works can be very effective initially they use accurate's usually requiring a relative to help technology but have moved to what calls these require a lot less knowledge from patients ideally all the work will now be carried out on tablet rather than a PC<br>Symptom checkers have been used theoretically this has improved access of UC consultant accurate kicks but all of these again have facilitated the dependence on our an answer from a general practitioner which is not necessary

**H**<br>**See Under T**	**training**		**MS**

J Changes and Potential

	Category	Themes	
**J**	**Changes & Potential**	**Shared Acceptance of principle of self care**	During the pandemic the public moved her becoming totally self caring by default<br><br>Amongst people and professionals there has been a significant move towards acceptance of home monitoring including oximeters and BP machines.<br><br>the ability the availability of the kit itself has also been a significant change having been discussed<br><br>previously<br><br>A positive way to encourage people to manage risk to help the increasing workload in general practice would be helpful in a way that was not deemed to be negative and rationing<br><br>Generally professional attitudes have not changed because they are battling with the same problems during the pandemic is pre-pandemic which is the nature of sarcoma care.<br><br>Some changes will be permanent this way to go before the culture of seeking help from the doctor can be changed
		**Use of online advice**	the pandemic itself has caused significant significant anxiety and there is a concern about googling you of professional contacts because of the range of confidence in online advice
		**Information**	**even as a GP the information she was given was minimal even such simple things that a temperature of 37.738 significant temperature for example**<br><br>**Specific messages have been developed to cut down bread and rice and to start and increase exercise**<br><br>There is a gap in the availability of good information about common symptoms for customers patients which could be built on the common the current interest

		**Change in professional role**	**pharmacists have a role in providing understanding resource for patients**<br><br>**signposting to with a like to get the best information help and assisting**<br><br>**example making hospital appointments, help with compliance aids, have been the deliveries for a wider range just pharmacists and in some cases providing shopping service**<br><br>Community Pharmacy services have come into their own particularly such elements as delivery services<br>vicar headmistress planning members and police officers involving regular meetings all found that they are dealing with the same clients heart sinks or high use of the same everyone<br><br>Fundamental change has been to give greater agency and autonomy to individuals to allow to self-care whilst also providing level professional fulfilment by Zoom<br><br>IT IS AN Opportunity that needs to be grasped immediately<br><br>Understanding of self-care has risen with the need for look after looking after relatives were even barn door things can be missed and the need for external advisers become clearer. Even with content health professions mistakes have been made
		**Change in Public approach**	Patients have been more willing to self-care particular using alternative medicine include I have edict medicine looking for other ways to manage their illnesses falling back on traditional treatments<br><br>Customers have become more responsible for their own self-care of the hygiene which is fundamental change knowing their own signs and symptoms to be concerned about monitoring as a fundamental change in the way people have behaved<br><br>Entirely sure that please have been picked up by pharmacists<br>Important things about people considering what’s important increasingly<br>deal with what people themselves who is important people have been interested in hobbies such as gardening what they like doing doing something that interests them such as going out and mindfulness and give the tools to help them<br>Benefits of moving to self-care is a first option is about people taking over control of their own lifestyle and taking responsibility for this

			The negative is what if they don't seek care when a problem is established<br><br>It's been a good thing that people have started to take care of themselves during lockdown many are now looking for other ways to continue this<br><br>As the pandemic progress people found ways of looking after themselves including new hobbies and interests for finding new ways of keeping themselves healthy<br><br>The implications are good that patients looking after help health preventing long-term conditions having healthy habits and spotting early changes
		**Self monitoring**	Getting patients to buy their own machines for monitoring blood pressure giving machines for diabetic monitoring has demonstrated a great willingness for people to do their own monitoring local schemes to give IPP machines have been very effective<br><br>Else oximetry has also been a means for patient empowerment and is used as an opportunity to ensure self-care messages are heard

K Motivation

		**Theme**	
**K**	**Motivation**	**Demography**	different for each term demographic
		**Agency**	good health
		**General**	People were SCARED and the only people available face to face was often the pharmacist
		**Self Management**	Understanding condition<br>take control to make your condition<br>better<br>The pharmacists have been any unique position to the pandemic as there remained the only healthcare professionals and those easy access and no appointments<br>people turn to pharmacist during the pandemic because of their accessibility<br>a lot of people sought advice<br>increasingly the discussions moved into the area of clinical advice<br>for example checking in with elderly isolating patients on a weekly basis

L Problems

		Theme	
L	Problems	Unmet need	We are now seeing particularly a rebound in the middle zone of the continuum with a massive demand from people who have been by default self caring and storing problems rather than perhaps dealing with them<br><br>A further problem has been the turn Covid patients tend to present data with more extensive disease because they have been missed is not want to bother professionals with a perception a GP surgeries are closed
		Alienation	The rise in telephone triage may well have led to this fact is satisfaction on both sides because the loss of the soft cues the empathy and the consideration of belief which may be lost in online consultations<br><br>More research is required around areas where self-care is far superior as there is a danger that encouraging self-care can be seen as rationing with a potential backlash. The sudden demand after lockdown is of great concern is the GP role so 02 fewer GPs prior to the pandemic<br><br>During Covid 19 with the move away from face-to-face consultations telephone consultations with people who may not know is been a process of alienation between the clinician and patient
		Access	Some people have been empowered some cancers may well have been missed<br><br>The family this means it is more difficult and to get support and they are unable to see the progress the family member before the return home is also more difficult the hospital to monitor progress<br><br>The elderly Bame population as a particular problem because they find it difficult to access resources<br><br>access to translators is a problem even when booking an appointment<br><br>Some patients have been shortchanged by the practices who have effectively shut the messages being don’t come here go home leaving patients to deal with their dependence on general practice which was not of their own making

		**Quality of Information**	Unfortunately GPs were saying go to your pharmacist for a cheque when they not negate the pharmacies at all and therefore information was inconsistent<br><br>Frontline it was time people suggested that the problem was over<br><br>Other frontliners been stressful was staff and pressure, particularly when practices close down effectively<br><br>Pharmacist were often turned to however when rules were changed and had no more information than anybody else and were not provided in a timely fashion therefore it's going to work pharmacist to receive information there also constantly asking about tiers and new now holiday travel<br><br>Even I use of the term viral infection tells the patient nothing the diagnosis should be ye seem to deal with other things time using made available of this<br><br>There was an issue about getting red flags dealt with when they were identified which was quite scary for pharmacists<br><br>It was felt this could not be left up to the patient to contact Dr and directory are is essential<br><br>CP CS services means that people are referred now to pharmacist for minor illness and is important to have a route for dealing with these in a red flag is identified
		**New relationship**	Changes will only be permanent if there is a change in GP training otherwise the same attitudes to Dr centrality will continue<br><br>Even the examination of patients in silence has been a problem and we need to be begin a dialogue with outpatients.
		**Training need**	I like to think the changes were permanent but simple education of patient is not effective E2 fundamental change to medical training<br><br>once the patient is through to you restriction of one problem per patient is produced<br><br>education the crucial<br><br>Self-care does not have its own domain many textbooks is not used in examinations investigations

			The use of the history in order to make a diagnosis with judicious use of examination is required but this will require changes in secondary care behaviour

**M**	**Benefits**		

N Attitudes

	Category	Theme	
**N**	**Attitudes**	**Extended Professional support for self care**	The positive the self-care will be permanent for instance home monitoring blood pressure and oximetry and understanding from the population of self-limiting issues self-illnesses do get better by themselves and a greater understanding of natural history of illnesses<br><br>People are now used to monitoring their own health including blood pressure and oximetry and diabetes and this is like to be a permanent change<br><br>The decreasing numbers are trivial consultations has been an obvious and permanent change

			Given the culture that has been a change which means that people are more likely to accept the change in medication with the matter insisted on before<br><br>These consultations will take time and it's important that this behaviour change is supported and funded<br><br>People are now more likely to see their pharmacist for trivial consultations but is likely to continue<br><br>Given the shortage of doctors is an opportune the grasped<br><br>Benefits of moving to self-care is a first option is about people taking over control of their own lifestyle and taking responsibility for this<br><br>The negative is what if they don't seek care when a problem is established<br><br>It's been a good thing that people have started to take care of themselves during lockdown many are now looking for other ways to continue this<br><br>Personally change in attitude is permanent because it gets one set up the day better able to Take care of myself<br><br>Many customers are taken a similar view<br><br>There has been a permanent change in attitudes on both sides and self-care has been embraced by both sides is now question seeing how much can to help everybody
		**Quality of Information and advice**	However the increasing demand demonstrates that people do not have complete confidence in the information they have obtained during the pandemic.<br><br>There has been confidence in the in NHS advice with confirmed approval of NHS opinion over such aspects of self isolation and vaccination an important element of this has been that messages are always prefaced with a common that people not be blocked from seeing the Dr

			A positive way to encourage people to manage risk to help the increasing workload in general practice would be helpful in a way that was not deemed to be negative and rationing
		**Opportunity**	This could be an opportunity
		**Boundaries of professional/self care**	In the balance between care and self-care must always be the opportunity to speak to a doctor who is known to have the evidence base for their decisions.<br><br>A feeling we are working towards some commonsense approach to self-care and a feeling that balances required between professionals and the people they serve in a feeling of justice<br><br>Customers have become more responsible for their own self-care of the hygiene which is fundamental change knowing their own signs and symptoms to be concerned about monitoring as a fundamental change in the way people have behaved<br><br>Entirely sure that please have been picked up by pharmacists<br><br>Fundamental change has been to give greater agency and autonomy to individuals to allow to self-care whilst also providing level professional fulfilment by Zoom<br><br>IT IS AN Opportunity that needs to be grasped immediately
		**Personal self care**	Individually Covid had such a severe effect on our constant changes in expectations and concerns and personal life changes that she had to realise she is not impervious his changes and while struggling the workers important that she the sake of others as well as herself sought help. Having said this later on<br><br>Doctors own experience of healthcare problems during the Covid pandemic has led to her understanding how health issues are treated poorly in the system performance is important to rely on self-care<br><br>GPs tend to be rather poor at taking own advice despite it being the biomedical year<br>Understanding of self-care has risen with the need for look after looking after relatives were even barn door things can be missed and the need for external advisers become clearer. Even with content health

			professions mistakes have been made and self-care therefore is also about making sure relatives get the care they need<br><br>Personally change in attitude is permanent because it gets one set up the day better able to Take care of myself<br><br>Many customers are taken a similar view

		**Themes**	
**P**	**Big Points**		1. There is a need to give people the confidence to self-care along with necessary support otherwise is a danger that people will feel abandoned rather than empowered.<br>2. SC has been everybody's job but nobody's responsibility<br>3. Some people have been empowered some cancers may well have been missed<br>4. GP – self-care is what patients do to help themselves before contacting you<br>5. Countries of origin make a significant difference attitudes to say self-care as in the rest of Europe<br>6. There is absolute need for information to be<br>7. digestible<br>8. comprehensible<br>9. to make sense for the individual<br>10. with specific advice on what to do next<br>11. Main motivation for patients to look after themselves<br>12. different for each term demographic<br>13. **Unfortunately access to treatment is too simple many people want medicine because that's the way they expect to be treated**<br>14. Perceptions of now 24/7 expect things to be fixedThe emphasis has been excess not accessAs long as people continue to encourage to have open access to trusted advisor there is no rational

			need to self-care even though this might have been the case during the pandemic. Why research things when you can ring the doctor or the lights on hospital 24 hours a day<br>15. Self-care is now means access to the fix which equals GP this has been a societal change told do it to rational thing to do therefore<br>16. NHS is seen as rescue service for victims<br>17. patient requires rescuing and the doctor is the rescuer<br>18. unfortunately hundred percent die third have no identifiable diagnosis and some of the secondary gain in their in this continuing<br>19. The antidote to this is fundamentally giving autonomy to the individual<br>20. IT IS AN Opportunity that needs to be grasped immediately<br>21. 90% of diagnosis are made on history and yet suggest that the patient needs to be examined in this very<br>22. Time is the biggest constraint<br>23. Changes will only be permanent if there is a change in GP training otherwise the same attitudes to Dr centrality will continue<br>24. There has been a permanent change in attitudes on both sides and self-care has been embraced by both sides is now question seeing how much can to help everybody

**S**<br>**See under technology - Duplicated**	**Self management**		People are now used to monitoring their own health including blood pressure and oximetry and diabetes and this is like to be a permanent change<br><br>Self-care fundamentally changing the people put their own thermometers pulse oximeters for heart rate as well as oximetry BP machines and even the simple things such as wife's and hand sanitiser are now part of the mainstay of self-care<br><br>Lateral flow tests in huge success though advice to pharmacist

		**Themes**	

**T**	**Training**	**Medical training**	How people are taught in GPs.<br>They have the helpers triad which is the exaggeration of responsibility, perfectionism, feelings of guilt<br>These lead to a feeling that if you do your stuff and diligent become a diligent student diligent medical school and didn't diligent secondary care doctor whose job is to exclude risk not manager this however when people are booked into general practice things change and risks cannot be excluded completely by have to be managed the victim versus rescuers as taught throughout training is no longer adequate<br>Self-care does not have its own domain many textbooks is not used in examinations investigations<br>The pandemic is an opportunity which needs to be grasped GP training is required
	**Reimubusement funding?**		
	**Lifestyle medince?**		
	**Competencies**		
	PS		Y done
	HH		Y done
	DM		Y done
	PB		Done
	HA		Ydone
	VR		Done
	MS		Done
	AS		
	AD		yDone
