# Supplementary material for: How has COVID-19 changed healthcare professionals’ attitudes to self-care? A mixed methods research study
Source: PLoS One. 2023 Jul 24;18(7):e0289067. doi: 10.1371/journal.pone.0289067 (PMC10365300; doi:10.1371/journal.pone.0289067)
Supplement: S3 File — (PDF) [file pone.0289067.s005.pdf]

Changes in the Attitudes and Practices of Health & Social Care Professionals with Respect to Self-Care as a Result of the COVID-19 Pandemic (CAPPS)

Study ID:

Name of Principal Investigator: Dr Austen El-Osta

Co-investigators: Dr Pete Smith
Mr Aos Alaa
Ms Immy Webber
Ms Eva Riboli-Sasco
Professor Azeem Majeed

© Imperial College of Science, Technology and Medicine

Crib Sheet Questions

1. **Thank you for taking the time to speak with me today. Please can I confirm if I have your consent to taking part in this interview? If you consent, your responses will be recorded on the electronic consent form I sent you.**

2. What are your views on the benefits of moving to self-care as a primary mode of care?

3. How do you personally feel about self-care? Has COVID-19 changed your attitudes?

4. How do you feel the COVID-19 pandemic has changed the landscape of care/self-care (e.g., delivery of care, willingness of patient to self-care)?

5. What do you believe to be the main motivations and/or barriers for your patients/clients to self-care (or for self-care as a primary mode of care)?

6. How has the advent of COVID-19 changed your and/or your patients/clients use of technology, specifically with regards to self-care. What are the main barriers/drivers?

7. Do you believe the changes seen in attitudes to self-care to be permanent? Why?

8. What do you believe are the implications?

© Imperial College of Science, Technology and Medicine
