# Supplementary material for: How has COVID-19 changed healthcare professionals’ attitudes to self-care? A mixed methods research study
Source: PLoS One. 2023 Jul 24;18(7):e0289067. doi: 10.1371/journal.pone.0289067 (PMC10365300; doi:10.1371/journal.pone.0289067)
Supplement: S4 File — (DOCX) [file pone.0289067.s006.docx]

**Consolidated criteria for reporting qualitative studies (COREQ): 32-item checklist**

Developed from:

Tong A, Sainsbury P, Craig J. Consolidated criteria for reporting qualitative research (COREQ): a 32-item checklist for interviews and focus groups. *International Journal for Quality in Health Care*. 2007. Volume 19, Number 6: pp. 349 – 357

| **No. Item** | **Guide questions/description** | **Reported on Page #** |
| --- | --- | --- |
| **Domain 1: Research team and reﬂexivity** |  |  |
| *Personal Characteristics* |  |  |
| 1. Inter viewer/facilitator | Which author/s conducted the inter view or focus group? | P8 Peter Smith |
| 2. Credentials | What were the researcher’s credentials? E.g. PhD, MD | Dr, GP P8 |
| 3. Occupation | What was their occupation at the time of the study? | P8 General Practitioner |
| 4. Gender | Was the researcher male or female? | Male P 8 |
| 5. Experience and training | What experience or training did the researcher have? | GP P 8 |
| *Relationship with participants* |  |  |
| 6. Relationship established | Was a relationship established prior to study commencement? | No |
| 7. Participant knowledge of the interviewer | What did the participants know about the researcher? e.g. personal goals, reasons for doing the research | Page 8  **Participants were told who the interviewer was, the purpose of the study, the length of time of the interview, where the data was stored and for how long. They were able to withdraw at any point, without giving a reason leading up to, or during the interview; advised not to answer questions they were uncomfortable with, without giving any explanations. Ethical had granted, participants reviewed the participant information sheet prior to giving their written informed consent to be involved.** |
| 8. Interviewer characteristics | What characteristics were reported about the inter viewer/facilitator? e.g. Bias, assumptions, reasons and interests in the research topic | Not reported on page |

| **Domain 2: study design** |  |  |
| --- | --- | --- |
| *Theoretical framework* |  |  |
| 9. Methodological orientation and Theory | What methodological orientation was stated to underpin the study? e.g. grounded theory, discourse analysis, ethnography, phenomenology, content analysis | Page 2, page 6 |
| *Participant selection* |  |  |
| 10. Sampling | How were participants selected? e.g. purposive, convenience, consecutive, snowball | Page 6 |
| 11. Method of approach | How were participants approached? e.g. face-to-face, telephone, mail, email | Page 6 |
| 12. Sample size | How many participants were in the study? | Page 12 |
| 13. Non-participation | How many people refused to participate or dropped out? Reasons? | **None** |
| *Setting* |  |  |
| 14. Setting of data collection | Where was the data collected? e.g. home, clinic, workplace | **Computer** |
| 15. Presence of non-participants | Was anyone else present besides the participants and researchers? | **No** |
| 16. Description of sample | What are the important characteristics of the sample? e.g. demographic data, date | Page 12 table 1 |
| *Data collection* |  |  |
| 17. Interview guide | Were questions, prompts, guides provided by the authors? Was it pilot tested? | S3- Interview Guide |
| 18. Repeat interviews | Were repeat inter views carried out? If yes, how many? | **No** |
| 19. Audio/visual recording | Did the research use audio or visual recording to collect the data? | Page 9 |
| 20. Field notes | Were ﬁeld notes made during and/or after the inter view or focus group? | S2 Interview Codes? |
| 21. Duration | What was the duration of the inter views or focus group? | Page 9 |
| 22. Data saturation | Was data saturation discussed? | Page 9 |
| 23. Transcripts returned | Were transcripts returned to participants for comment and/or correction? | **No** |
| **Domain 3: analysis and ﬁndings** |  |  |
| *Data analysis* |  |  |
| 24. Number of data coders | How many data coders coded the data? | **One (The co-author)** |
| 25. Description of the coding tree | Did authors provide a description of the coding tree? | S2 Interview Codes |
| 26. Derivation of themes | Were themes identiﬁed in advance or derived from the data? | S2 Interview Codes **Themes were derived from the data** |
| 27. Software | What software, if applicable, was used to manage the data? | **Microsoft Word, Excel & Qualtrics** |
| 28. Participant checking | Did participants provide feedback on the ﬁndings? | **No** |
| *Reporting* |  |  |
| 29. Quotations presented | Were participant quotations presented to illustrate the themes/ﬁndings? Was each quotation identiﬁed? e.g. participant number | Pages 16-22 |
| 30. Data and ﬁndings consistent | Was there consistency between the data presented and the ﬁndings? | Page 31  **Yes** |
| 31. Clarity of major themes | Were major themes clearly presented in the ﬁndings? | Page 23-30  **Yes** |
| 32. Clarity of minor themes | Is there a description of diverse cases or discussion of minor themes? | Page 38  **Yes** |
